# Serum sclerostin and adverse outcomes in elderly patients with stable coronary artery disease undergoing percutaneous coronary intervention

**DOI:** 10.1007/s40520-019-01393-2

**Published:** 2019-11-01

**Authors:** Wuyang He, Chunqiu Li, Qingwei Chen, Tingting Xiang, Peng Wang, Jun Pang

**Affiliations:** Department of Geriatric Cardiology, The Second Affiliated Hospital of Chongqing Medical University, No 76 Linjiang Road, Chongqing, 400010 China

**Keywords:** Sclerostin, Stable coronary artery disease, Percutaneous coronary intervention, Elderly

## Abstract

**Background:**

Recently, sclerostin, a bone-derived protein, has been shown to play a key role in atherosclerosis progression. However, few studies have investigated the influence of sclerostin on cardiovascular disease prognosis. We investigated the relationship between serum sclerostin levels and adverse outcomes in elderly patients with stable coronary artery disease (SCAD) who were undergoing percutaneous coronary intervention (PCI).

**Methods:**

We enrolled 310 elderly SCAD patients who underwent PCI in this study and followed them 3 years. According to the median serum sclerostin levels, subjects were stratified into a low sclerostin (low scl) group (*n* = 144) and a high sclerostin (high scl) group (*n* = 166). Time-to-event analyses were performed with the Kaplan–Meier method. Associations between sclerostin levels and main adverse cardiovascular and cerebrovascular events (MACCEs) and mortality were evaluated by Cox multivariate regression analysis. The prognostic power of predictive models was verified by the concordance index and receiver operating characteristic curve analysis.

**Results:**

The high scl group had a significantly higher MACCE-free rate and better survival than the low scl group. Serum sclerostin was an independent predictor and could improve the prognostic power for adverse outcomes. In addition, serum sclerostin levels were significantly associated with bone turnover markers, a lower presence of multivessel disease and a lower CCS angina class.

**Conclusions:**

Serum sclerostin is a prognostic parameter for predicting and intervening in the adverse outcomes of elderly SCAD patients undergoing PCI, which may be explained by its potential role in the bone–vascular axis.

**Electronic supplementary material:**

The online version of this article (10.1007/s40520-019-01393-2) contains supplementary material, which is available to authorized users.

## Introduction

Coronary heart disease (CHD) has become the leading cause of death worldwide, increasing both mortality and the risk of disability in the elderly population. Several studies have reported that bone loss may increase the risk of CHD [1, 2], and its underlying mechanism could be explained by the “bone–vascular calcification paradox”. This phenomenon has been defined as the coincidence of bone loss and vascular calcification [3], revealing the common pathophysiological process between atherosclerosis and osteoporosis.

The canonical Wnt-signaling pathway promotes bone formation by stimulating the differentiation of mesenchymal stem cells (MSCs) to osteoblasts and attenuating the differentiation of osteoclasts [4, 5]. Since osteogenic differentiation of MSCs and pericytes also occurs in the process of vascular calcification [6], the canonical Wnt-signaling pathway may be involved in the link between atherosclerosis and bone loss. Sclerostin, an antagonist in the canonical Wnt-signaling pathway, can inhibit ectopic calcification in the vasculature, exerting its potential protective role by attenuating the progression of atherosclerosis [7]. The role of sclerostin can also be supported by the overexpression of sclerostin in calcified aortic vascular tissue [8]. Thus, investigating the influence of circulating sclerostin on the prognosis of CHD could provide a new target for improving survival and lifespan in elderly populations.

Recent data have demonstrated that circulating sclerostin levels are positively correlated with better survival in patients undergoing hemodialysis [9]. Nevertheless, other studies have shown that higher serum sclerostin levels increase all-cause mortality in patients with chronic kidney disease (CKD) [10]. Despite these contradictory findings, few studies have investigated the relationship in patients with normal renal function. Therefore, we followed a cohort of elderly stable coronary artery disease (SCAD) patients without CKD to assess the relationships between serum sclerostin levels and adverse outcomes after percutaneous coronary intervention (PCI).

## Materials and methods

### Participants and study design

A total of 310 elderly SCAD patients who underwent PCI at the Department of Geriatric Cardiology of the Second Affiliated Hospital of Chongqing Medical University (CQMU) between Jan 2014 and Dec 2015 were enrolled in this study. Participants were stratified into 2 groups according to the median value of serum sclerostin levels: those with low serum sclerostin levels (the low scl group) and those with high sclerostin levels (the high scl group). All subjects satisfied the following inclusion criteria: age ≥ 65 years, a diagnosis of SCAD [11], eligibility for PCI met, and willingness to participate in the study and sign a written informed consent form. The exclusion criteria included (1) a recent presence of acute coronary syndrome within 3 months; (2) PCI within 3 months or had a previous or planned CABG; (3) left main coronary artery disease, left ventricular ejection fraction < 50% as assessed by echocardiography, or Canadian Cardiovascular Society (CCS) angina class IV; (4) an eGFR < 45 mL/min/1.73 m^2^; (5) a diagnosis of infectious or autoimmune disease or current use of an anti-inflammatory treatment; (6) a history of hepatic insufficiency, significant valvular disease, CKD, a thyroid disorder or a malignant tumor; or (7) current treatment with drugs that could affect bone metabolism and serum sclerostin levels, such as calcium, vitamin D, thyroid hormone, thiazolidinedione, warfarin, or aspirin.

Our study was performed with the approval of the ethics committee of the Second Affiliated Hospital of CQMU, and informed consent was obtained from all participants.

### Clinical analysis

At baseline, age, sex, alcohol consumption, smoking habit, history of diseases, and medication usage history were collected through a specific questionnaire. Height and weight were measured by standardized methods. BMI was calculated using the weight divided by the square of the height. Blood pressure was measured using a standard mercury sphygmomanometer (12 cm long and 35 cm wide) after letting the subject rest for more than 5 min. The blood pressure for each subject was measured twice, and the mean value was recorded. The bone mineral density (BMD) of all subjects was measured at the lumber spine(LS) and femoral neck(FN) by dual energy X-ray absorptiometry (GE Lunar iDNA, America).

Frailty status was assessed using the frailty phenotype (FP) scale [12]. The presence of more than three items on the scale was defined as frailty.

The physical activity of all subjects was measured by the Chinese version of the International Physical Activity Questionnaire (IPAQ) short form, which has been validated to be accurate in the Chinese population [13]. Based on intensity, physical activity was classified as vigorous activity (VA), moderate activity, or walking and was evaluated by the frequency and duration of physical activity in the previous 7 days [14].

### Baseline biomedical measurements

Overnight venous blood samples were collected on the morning of PCI. Biochemical parameters included traditional cardiovascular risk factors [such as levels of triglyceride (TG), total cholesterol (TC), LDL-cholesterol (LDL-c), HDL-cholesterol (HDL-c), hypersensitive C-reactive protein (hs-CRP), glycated hemoglobin (HbA1c), creatine, etc.] and bone turnover markers (such as levels of parathyroid hormone (PTH), 25-hydroxyvitamin D (25(OH)D), bone alkaline phosphatase (BALP), N-terminal propeptide of type I procollagen (PINP), N-terminal midfragment of osteocalcin (N-MID osteocalcin), and C-terminal cross-linking telopeptide of type I collagen (β-CTX)] were immediately measured by standard biochemical methods. Moreover, all serum samples were stored at – 80 °C for the measurement of sclerostin. According to the manufacturer’s instructions, serum levels of sclerostin were measured by ELISA (DY1406, R&D SYSTEMS, America). The limit of detection was 41.5 pg/mlL. The intra- and interassay variability were both lower than 10%.

### CAD severity

Coronary angiography was performed using the Judkins technique via the radial artery. CAD severity was then evaluated using the SYNTAX score by two experienced interventional cardiologists who were not aware of the clinical results of the patients. SYNTAX score calculator 2.1 (www.syntaxscore.com) was used to measure the SYNTAX score for all coronary lesions, which was > 50% diameter stenosis in a vessel > 1.5 mm.

### Follow-up and outcome analysis

Follow-up with all subjects was achieved via telephone interview or was based on medical records obtained from the Second Affiliated Hospital of CQMU at 1 month, 6 months, 1 year, 2 years, and 3 years after PCI. The presence of main adverse cardiovascular and cerebrovascular events (MACCEs) (including all-cause death, stroke, myocardial infarction (MI) and repeat revascularization) was regarded as the primary endpoint. The prespecified secondary endpoints were the individual MACCE components and cardiovascular mortality.

### Definitions

Hypertension was defined based on a systolic blood pressure ≥ 140 mmHg, a diastolic blood pressure ≥ 90 mmHg or the use of antihypertension medication [15]. Type 2 diabetes mellitus (T2DM) was defined based on a level of HbA1c ≥ 6.5% or on a fasting glucose level ≥ 126 mg/dL obtained on two separate days or on the use of antidiabetic medication. Osteoporotic (OP) patients were diagnosed with prior fragility fractures or densitometry, defined by a T-score of − 2.5 or less. Peripheral artery disease was diagnosed according to the ACC/AHA guidelines [16]. Myocardial infarction and stroke were defined based on ischemic symptoms and clinical guidelines [17, 18].

### Statistical analysis

The Kolmogorov–Smirnov test was used to test the normality of continuous variables. Normally distributed variables between two groups were compared by Student’s *t* test. The Mann–Whitney *U* test was used to compare skewed variables. The data are shown as counts with percentages for qualitative values and as the means ± standard deviations (SDs) or medians with interquartile ranges for quantitative values. The *χ*^2^ test was used to compare categorical variables, which are presented as percentages. The linear regression analysis was used to assess the association between serum sclerostin and other clinical parameters. The Kaplan–Meier method was used to perform time-to-event analyses, which were compared between patients in the low scl group and the high scl group. Multivariate Cox regression analysis was used to evaluate the relationships between the baseline characteristics and adverse outcomes. The discrimination and predictive ability of the predictive model with or without sclerostin was tested using the concordance index (C-index) and receiver operating characteristic (ROC) curve analysis. IBM SPSS Statistics version 25.0, R version 3.6.1 for Windows, and GraphPad Prism 6 were used to analyze the data, and *p* values < 0.05 were considered significant.

## Results

### Baseline characteristics

All subjects were stratified into two groups according to the median value of serum sclerostin levels (179.5 pg/mL). The baseline characteristics are shown in Table 1. Compared with the low scl group, the high scl group had a lower prevalence of osteoporosis(42.6% vs 73.6%, *p* < 0.001), a higher BMD of the FN (0.9 vs 0.8 g/cm^3^, median, *p* < 0.001), a higher BMD of the LS (1.0 vs 0.9 g/cm^3^, median, *p* = 0.001), a lower rate of statin use (1.2% vs 6.3%, *p* = 0.017), and lower CCS class ratings (1.0 vs 1.0, median, *p* < 0.001). Regarding the CAD severity and angiographic characteristics, the differences between the two groups did not reach significance (*p* > 0.05).

**Table 1:** Baseline characteristics of the low and high sclerostin groups

Characteristic	Low scl group (*n* = 144)	High scl group (*n* = 166)	*p* value
*Conventional cardiovascular disease risk factors*			
Age, years	75 (67–83)	77 (69–83)	0.271
Male sex, %	64 (44.4)	72 (43.6)	0.886
Current smokers, %	25 (17.4)	39 (23.5)	0.183
Alcohol, %	20 (13.9)	29 (17.5)	0.389
Hypertension, %	96 (66.7)	101 (60.8)	0.288
Diabetes, %	53 (36.8)	57 (34.3)	0.651
Osteoporosis, %	106 (73.6)	76 (45.8)	< 0.001
PAD, %	15 (10.4)	14 (8.4)	0.550
History of MI, %	5 (3.5)	2 (1.2)	0.705
History of PCI, %	23 (16.0)	24 (14.5)	0.711
Systolic BP, mmHg	130 (119.3–136.0)	127 (117.8–136.0)	0.472
Diastolic BP, mmHg	75 (68–80)	72 (67–80)	0.246
BMI (kg/m^2^)	23.0 (20.8–24.9)	23.3 (20.8–25.5)	0.378
SCr (µmol/L)	71.6 (58.7–89.6)	72.0 (59.2–86.4)	0.999
HbA1c, %	6.1 (5.7–6.7)	6.2 (5.9–6.7)	0.064
TG, mmol/L	1.2 (0.8–1.9)	1.3 (1.0–1.8)	0.311
TC, mmol/L	4.5 ± 1.1	4.5 ± 1.0	0.994
LDL–C, mmol/L	2.5 ± 0.9	2.5 ± 0.8	0.886
HDL–C, mmol/L	1.2 ± 0.3	1.2 ± 0.3	0.774
CCS class	1.0 (1.0–2.0)	1.0 (1.0–1.0)	< 0.001
Frailty, %	37 (25.7)	54 (32.5)	0.187
VA, %	15 (10.4)	17 (10.2)	0.960
*Bone turnover markers*			
PTH, pg/mL	32.7 (21.8–45.6)	32.7 (20.1–52.7)	0.828
CT, pg/mL	1.8 (1.2–2.6)	1.8 (1.2–3.1)	0.120
25 (OH)D, ng/mL	14.5 (9.7–22.5)	16.7 (10.8–28.2)	0.143
BALP, µg/L	10.2 (8.2–13.1)	9.8 (8.0–12.3)	0.320
PINP, µg/L	35.5 (26.4–46.0)	36.3 (24.7–49.8)	0.807
β-CTX, µg/L	0.4 (0.2–0.5)	0.4 (0.2–0.5)	0.369
N-MID osteocalcin, µg/L	16.4 (11.7–24.2)	15.8 (11.7–21.2)	0.192
BMD (FN), g/cm3	0.8 (0.6–0.9)	0.9 (0.7–1.0)	< 0.001
BMD (LS), g/cm3	0.9 (0.8–1.1)	1.0 (0.9–1.2)	0.001
*Angiographic characteristics*			
SYNTAX scores ≥ 33, %	15 (10.4)	13 (7.8)	0.428
*Target vessels*			
LAD, %	89 (61.8)	102 (61.4)	0.948
LCX, %	48 (33.3)	58 (34.9)	0.766
RCA, %	60 (41.7)	66 (39.8)	0.733
Multivessel disease, %	17 (11.8%)	27 (16.3)	0.262
PCI failure, %	4 (2.8)	5 (3.0)	0.902
Total occlusion, %	71 (49.3)	85 (51.2)	0.739
Stent placement, %	130 (90.3)	152 (91.6)	0.693
*Medication*			
Platelet inhibitor, %	116 (80.6)	132 (79.5)	0.820
Beta blocker, %	42 (29.2)	45 (27.1)	0.687
Statin, %	9 (6.3)	2 (1.2)	0.017
RAS blocker, %	69 (47.9)	80 (48.2)	0.961

*PAD* peripheral artery disease, *MI* myocardial infarction, *PCI* percutaneous coronary intervention, *BP* blood pressure, *BMI* body mass index, *SCr* serum creatine, *HbA1c* glycated hemoglobin, *TG* triglycerides, *TC* total cholesterol, *LDL-C* low-density lipoprotein cholesterol, *HDL-C* high-density lipoprotein cholesterol, *CCS* Canadian Cardiovascular Society, *VA*: vigorous activity, *CT* calcitonin, 25(OH)D 25-hydroxyvitamin D, *BALP* bone alkaline phosphatase, *PINP* propeptide of type I procollagen, *β-CTX* C-terminal cross-linked telopeptide, *N-MID* osteocalcin N-terminal midfragment of osteocalcin, *BMD* bone mineral density, *FN* femoral neck, *LS* lumber spine, *LAD* left anterior descending artery, *LCX* left circumflex branch coronary artery, *RCA* right coronary artery

### The association of sclerostin with adverse outcomes after PCI

**Fig. 1:**
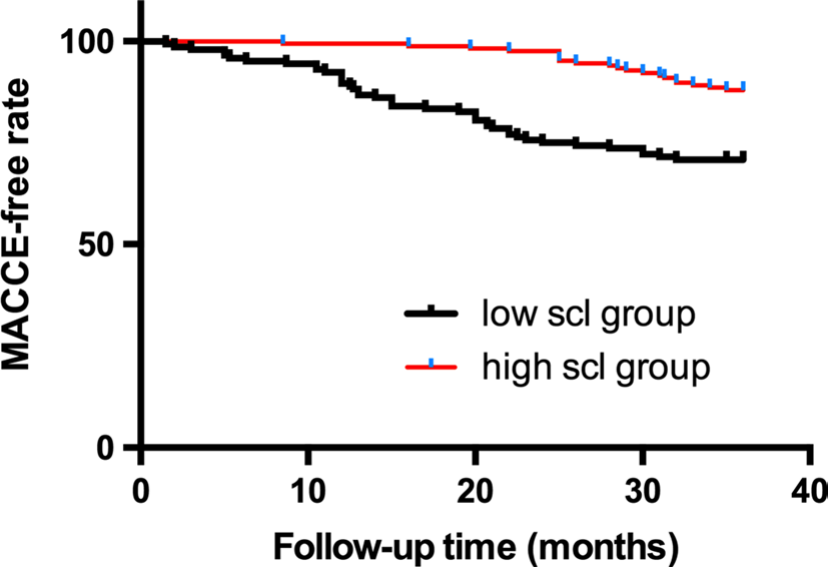
Kaplan–Meier survival curves of the MACCE-free rate for the low scl group and the high scl group (log-rank *p* < 0.001)

**Fig. 2:**
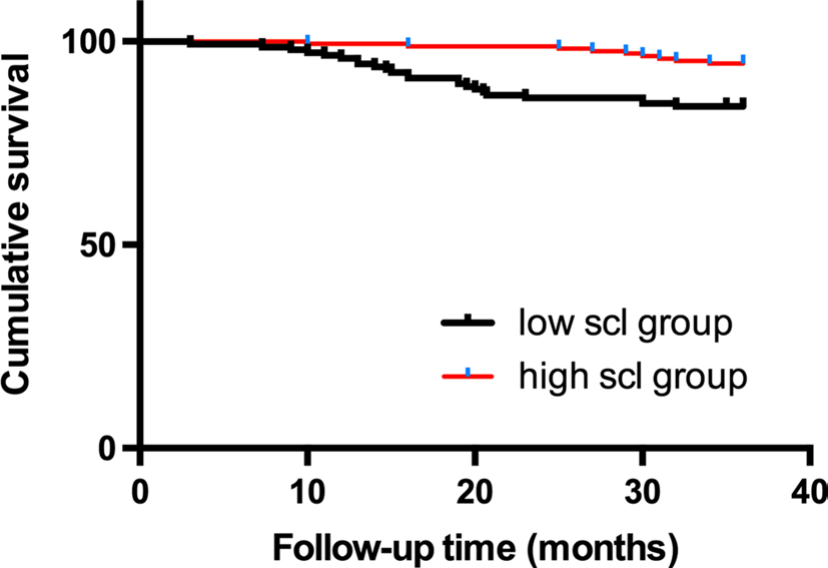
Kaplan–Meier survival curves of all-cause mortality for the low scl group and the high scl group (log-rank *p* = 0.002)

**Fig. 3:**
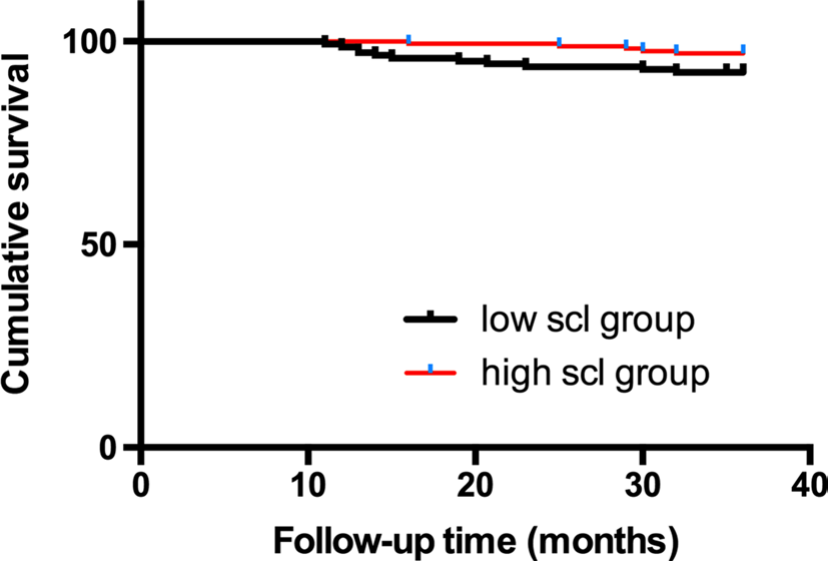
Kaplan–Meier survival curves of cardiovascular mortality for the low scl group and high scl group (log-rank *p* = 0.06)

During the 3-year follow-up period, one patient was lost during follow-up, 62 patients developed at least one MACCE, and 32 patients died, of whom 14 patients’ deaths were attributed to cardiovascular mortality. The time-to-event analyses were performed via the Kaplan–Meier method. The comparison between the low scl group and the high scl group was conducted using the log-rank test. The high scl group had a significantly lower presence of MACCEs (Fig. 1), a lower risk of MI, repeat revascularization, and stroke (Online Figs. 4, 5, 6) and better survival of all-cause mortality (Fig. 2) than the low scl group (all *p* < 0.05). There was no difference in cardiovascular mortality (Fig. 3) between the two groups.

In the multivariate Cox regression analysis (Table 2, Models 1–3), serum sclerostin was an independent predictor for MACCEs (HR = 0.456, 95% CI 0.245–0.848, *p* = 0.013). Among the components of MACCEs, serum sclerostin was independently associated with the components MI (HR = 0.369, 95% CI 0.156–0.871, *p* = 0.023) and all-cause mortality (HR = 0.297, 95% CI 0.117–0.752, *p* = 0.010) rather than stroke and repeat revascularization after adjusting for conventional cardiovascular risk factors, CAD severity, angiographic characteristics, and medication. However, serum sclerostin was not an independent parameter associated with cardiovascular mortality.

**Table 2:** Relationship between serum sclerostin levels and adverse outcomes in Cox multivariate regression analysis

Outcome	HR	95% CI	*p* value
*Model 1*			
MACCEs	0.461	0.252–0.844	0.012
All-cause mortality	0.342	0.143–0.820	0.016
MI	0.464	0.212–1.016	0.055
Stroke	1.417	0.478–4.200	0.530
Repeat revascularization	0.498	0.266–0.931	0.029
Cardiovascular mortality	0.531	0.104–2.701	0.446
*Model 2*			
MACCEs	0.466	0.254–0.854	0.014
All-cause mortality	0.342	0.143–0.822	0.016
MI	0.472	0.215–1.036	0.061
Stroke	1.582	0.520–4.816	0.419
Repeat revascularization	0.499	0.266–0.935	0.030
Cardiovascular mortality	0.525	0.103–2.680	0.439
*Model 3*			
MACCEs	0.456	0.245–0.848	0.013
All-cause mortality	0.297	0.117–0.752	0.010
MI	0.369	0.156–0871	0.023
Stroke	1.635	0.522–5.123	0.399
Repeat revascularization	0.617	0.318–1.195	0.152
Cardiovascular mortality	0.408	0.066–2.543	0.337

Model 1 is adjusted for the conventional cardiovascular disease risk factors, the presence of osteoporosis, BMD(FN), BMD(LS), angiographic characteristics, and the parameters with p <0.05 in the univariate analysis. Model 2 is further adjusted for the presence of total occlusion based on model 1. Model 3 is additionally adjusted for the presence of total occlusion and medication based on model 1

*MACCEs* main adverse cardiac and cerebral events, *MI* myocardial infarction, *C-index* concordance index, *AUC* area under the curve

As the independent factors in the multivariate Cox model were further adopted into the predictive model for adverse outcomes, we compared the discriminatory and predictive power of each predictive model with or without sclerostin. The C-index and AUC are presented in Online Table 4. Respectively, the predictive models with sclerostin had higher accuracy than the models excluding sclerostin in the prediction of MACCEs (C-index 0.867 vs 0.857), the component of all-cause mortality (C-index 0.773 vs 0.713) and MI (C-index 0.856 vs 0.824). The inclusion of sclerostin in the predictive model could improve the prognostic power for MACCEs (AUC 0.895 vs 0.888), all-cause mortality (AUC 0.778 vs 0.723) and MI (AUC 0.870 vs 0.839).

### The baseline parameters associated with serum sclerostin levels

In the multivariate linear regression analysis, after adjusting for conventional cardiovascular risk factors, angiographic characteristics, and the use of medication, N-MID osteocalcin(*β* = − 0.357, *p* < 0.001), β-CTX (*β* = 0.200, *p* = 0.012), PINP(*β* = 0.207, *p* = 0.006), CCS class (*β* = − 0.160, *p* = 0.017), PAD(*β* = 0.132, *p* = 0.042), and multivessel disease(*β* = − 0.223, *p* = 0.005) remained significantly associated with serum sclerostin levels (Table 3).

**Table 3:** Correlation analysis between sclerostin levels and clinical parameters

Variable	*β*	*T*	*p* values
N-MID osteocalcin	− 0.357	− 3.729	< 0.001
β-CTX	0.200	2.529	0.012
PINP	0.207	2.777	0.006
CCS class	− 0.160	− 2.409	0.017
PAD	0.132	2.040	0.042
Multivessel disease	− 0.223	− 2.845	0.005

*N-MID osteocalcin* N-terminal midfragment of osteocalcin, *β-CTX* C-terminal cross-linked telopeptide, *PINP* propeptide of type I procollagen, *CCS* Canadian Cardiovascular Society, *PAD* peripheral artery disease

## Discussion

The important role of sclerostin in the kidney–bone–vascular axis has been increasingly reported. However, whether serum sclerostin is involved in extraosseous and intraosseous mineralization in patients with normal renal function remains to be investigated, and the association of sclerostin with the adverse outcomes of PCI is scarcely discussed. Our study revealed that elderly SCAD patients with high sclerostin levels had a significantly lower incidence rate of MACCEs and better survival after PCI. More importantly, we showed an independent predictive role of serum sclerostin for the risk of MACCEs, all-cause mortality, and MI, even after adjusting for other confounding factors. The inclusion of sclerostin could add to the prognostic power in predicting adverse outcomes. In addition, higher serum sclerostin levels were associated with higher PINP levels, lower CCS classes of angina, and a lower presence of multivessel disease.

In our study, patients with high sclerostin levels had a significantly lower risk of MACCEs and longer survival, which is consistent with the association between higher circulating sclerostin levels and improved survival reported by previous studies [19, 9]. Our results disclose that sclerostin could be an intervening target to reduce the risk of MACCEs and improve the survival of elderly SCAD patients after PCI. Recent data indicate that antisclerostin monoclonal antibody (Scl-Ab) therapy promotes bone formation and inhibits resorption [20], which may otherwise increase the risk of cardiovascular events [21, 22], since downregulating circulating sclerostin may induce osteogenic differentiation of pericytes and MSCs [5]. Considering that Scl-Ab is an emerging approach to metabolic bone disease, more strategies are needed to attenuate the adverse effects of Scl-Ab in elderly SCAD patients.

The current study further showed that the serum sclerostin level was an independent predictor of MACCEs and the component MI. Moreover, serum sclerostin could also enhance the accuracy and predictive ability of the predictive model, implying the evaluation of baseline serum sclerostin levels as a reliable method to monitor adverse outcomes in elderly SCAD patients undergoing PCI. Considering the strongly indicative role of vascular calcification in cardiovascular events, serum sclerostin may add prognostic power for MACCEs and its component because of its role in regulating vascular calcification [23]. While the vasculature is the most highly mineralized structure second to the skeleton, the newly developed vascular calcification can be attributed to ectopic mineralization of the vascular wall, which is activated by the canonical Wnt-signaling pathway [24, 25]. As sclerostin protein was detected via human aortic extraction using proteomic methodology [26], elevated SOST expression during osteocytic differentiation of murine vascular smooth muscle cells and increased expression of sclerostin protein in the medial calcification of mouse aorta suggests a protective role of sclerostin in halting vascular calcification via interacting with LRP4/5/6 and inhibiting the canonical Wnt pathway [25]. This finding was further verified by two different mouse models, showing that overexpression of sclerostin could prevent aortic rupture and atherosclerosis progression in the mice, decreasing the expression of osteogenic markers such as osteoprotegerin and osteopontin in aortic tissues [27].

Considering that serum sclerostin was positively associated with bone formation marker(PINP) and negatively associated with MACCEs and surrogate markers of CAD severity (including CCS angina class and presence of multivessel disease) in our study, sclerostin may be speculated to mediate the bone–vascular axis. Since serum sclerostin is secreted mainly by mature osteocytes [28], the downregulating circulating sclerostin could parallel the bone mass loss and impaired bone metabolism of elderly people, which is also supported by the higher BMD values in high scl group in this study. Bone metabolism dysfunction attributed to aging may initiate the loss of bone mass and downregulation of serum sclerostin levels, accelerating the atherosclerosis process in elderly SCAD patients undergoing PCI. Although the causal relationship still needs further verification, sclerostin tends to affect cardiovascular outcomes by mediating the dynamic process of skeletal anabolism and atherosclerosis progression based on previous and current data. Thus, enhancing bone formation and bone mass could be beneficial for improving serum sclerostin and attenuating the adverse outcomes after PCI.

Interestingly, despite the predictive ability of sclerostin for all-cause mortality in the current study, serum sclerostin was not associated with cardiovascular mortality, which differs from the independent relationship between sclerostin and cardiovascular mortality revealed by a previous result [29]. The discrepancy of the results may be attributable to the difference in demographic characteristics, considering that patients with chronic kidney disease were enrolled in the previous study and we focused on elderly SCAD patients with normal renal function. Another mechanism could be involved in the regulation of sclerostin in all-cause mortality in addition to its protective effect on the cardiovascular system. Since systemic inflammation contributes to higher mortality in elderly patients, it is evident that overexpression of sclerostin downregulated the circulating levels of inflammatory factors in transgenic mice [27]. Clinical data also support the negative association of serum sclerostin levels with inflammation and structural damage [30]. Considering that skeletal muscle mass is a predictor of mortality of patients [31, 32], lean muscle mass was decreased in SOST-deficient mice, and a negative relationship of serum sclerostin and skeletal muscle mass was also reported in healthy subjects without diabetes [33]. Given these results, we propose that sclerostin may affect all-cause mortality in elderly SCAD patients by regulating systemic inflammation and skeletal muscle mass.

There are some limitations to this study. First, a single-centered observational study might not be able to verify the causal relationship between sclerostin and adverse outcomes and show the molecular mechanism of the bone–vascular axis. In addition, further multicentered follow-up studies are also needed to illustrate the predictive role of sclerostin for adverse outcomes in patients with normal renal function. Second, the study sample was comparatively small. However, we have several strengths. First, as far as we know, the current study is the first to investigate the relationship between serum sclerostin levels and adverse outcomes of elderly SCAD patients undergoing PCI, which provides clinical basis for the further mechanistic insights. Second, the exhaustive measurement of variables (such as conventional cardiovascular risk factors, bone turnover markers, BMD, and angiographic characteristics) associated with patient outcomes after PCI were included in this study.

## Conclusions

In conclusion, we confirmed, for the first time, that higher serum sclerostin levels are associated with better 3-year outcomes after PCI in elderly SCAD patients. Moreover, serum sclerostin is an independent predictive parameter that improves the prognostic power for the risk of MACCEs, MI, and all-cause mortality, implying that serum sclerostin may be a new target for predicting and intervening in the adverse outcomes of elderly SCAD patients. The relationship between serum sclerostin levels and adverse outcomes can be explained by the implication of sclerostinin the bone–vascular axis. Nevertheless, the underlying molecular mechanism merits further investigation.

## Electronic supplementary material

Below is the link to the electronic supplementary material.

Supplementary material 1 (DOCX 382 kb)
