# Supplementary material for: Serum sclerostin and adverse outcomes in elderly patients with stable coronary artery disease undergoing percutaneous coronary intervention
Source: Aging Clin Exp Res. 2019 Nov 1;32(10):2065–72. doi: 10.1007/s40520-019-01393-2 (PMC7532957; doi:10.1007/s40520-019-01393-2)
Supplement: Supplementary file 1 — Supplementary material 1 (DOCX 382 kb) [file 40520_2019_1393_MOESM1_ESM.docx]

**Appendix**

**
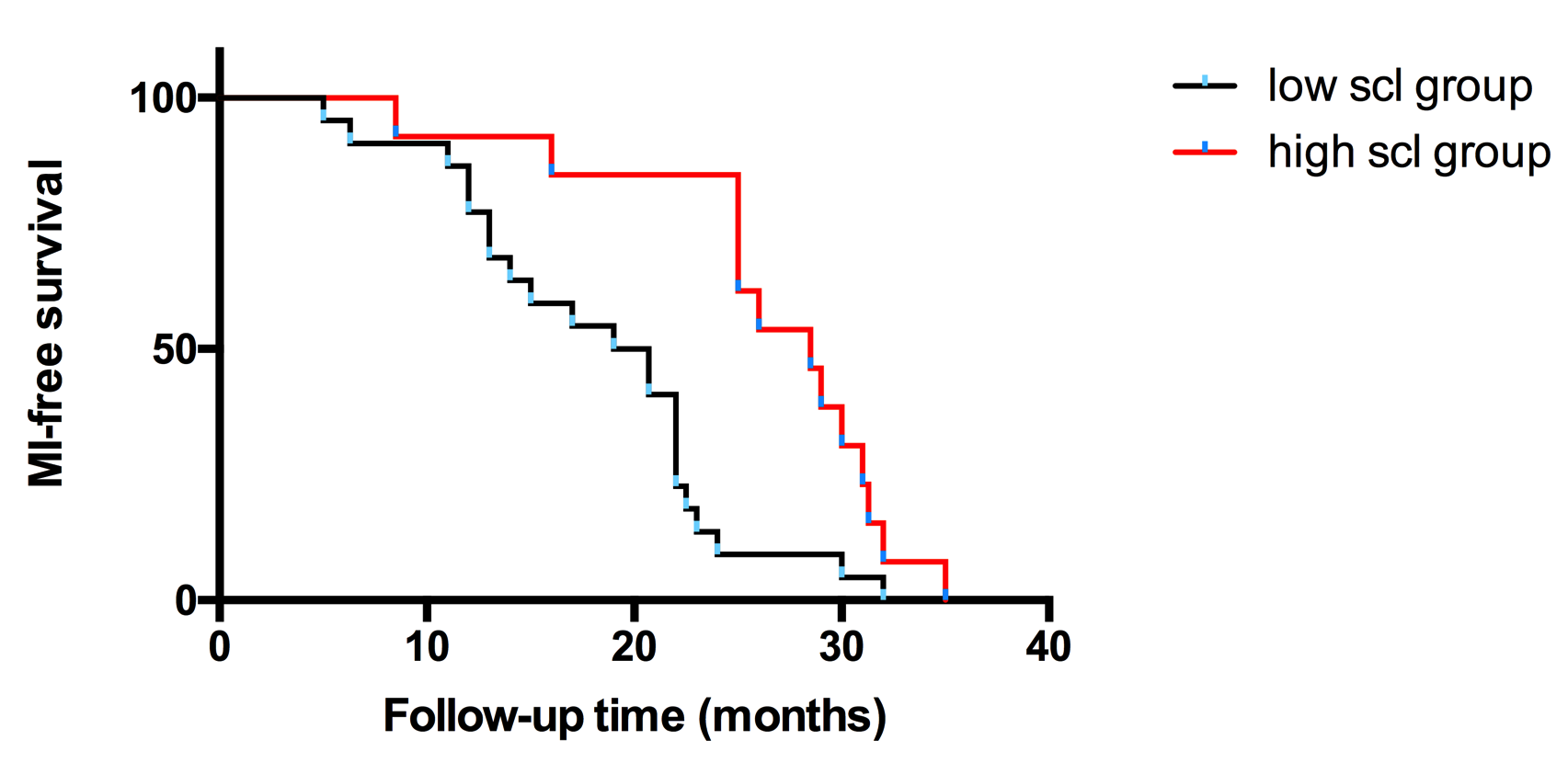
**

**Fig. 4** Kaplan-Meier survival curves of the MI-free rate for the low scl group and the high scl group(log-rank *p*=0.002). MI, myocardial infarction.


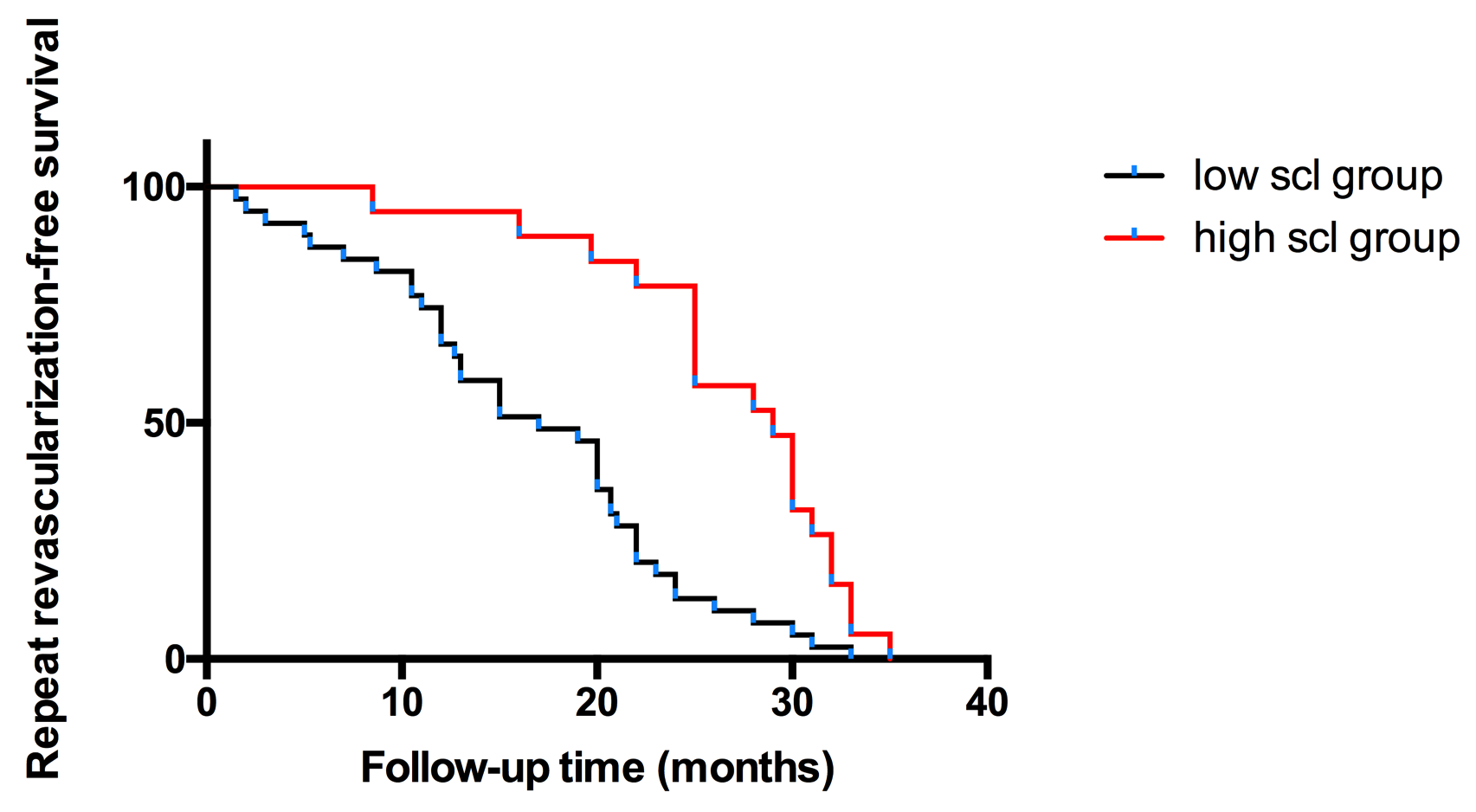


**Fig. 5** Kaplan-Meier survival curves of the repeat revascularization-free rate for the low scl group and the high scl group(log-rank *p*<0.001).


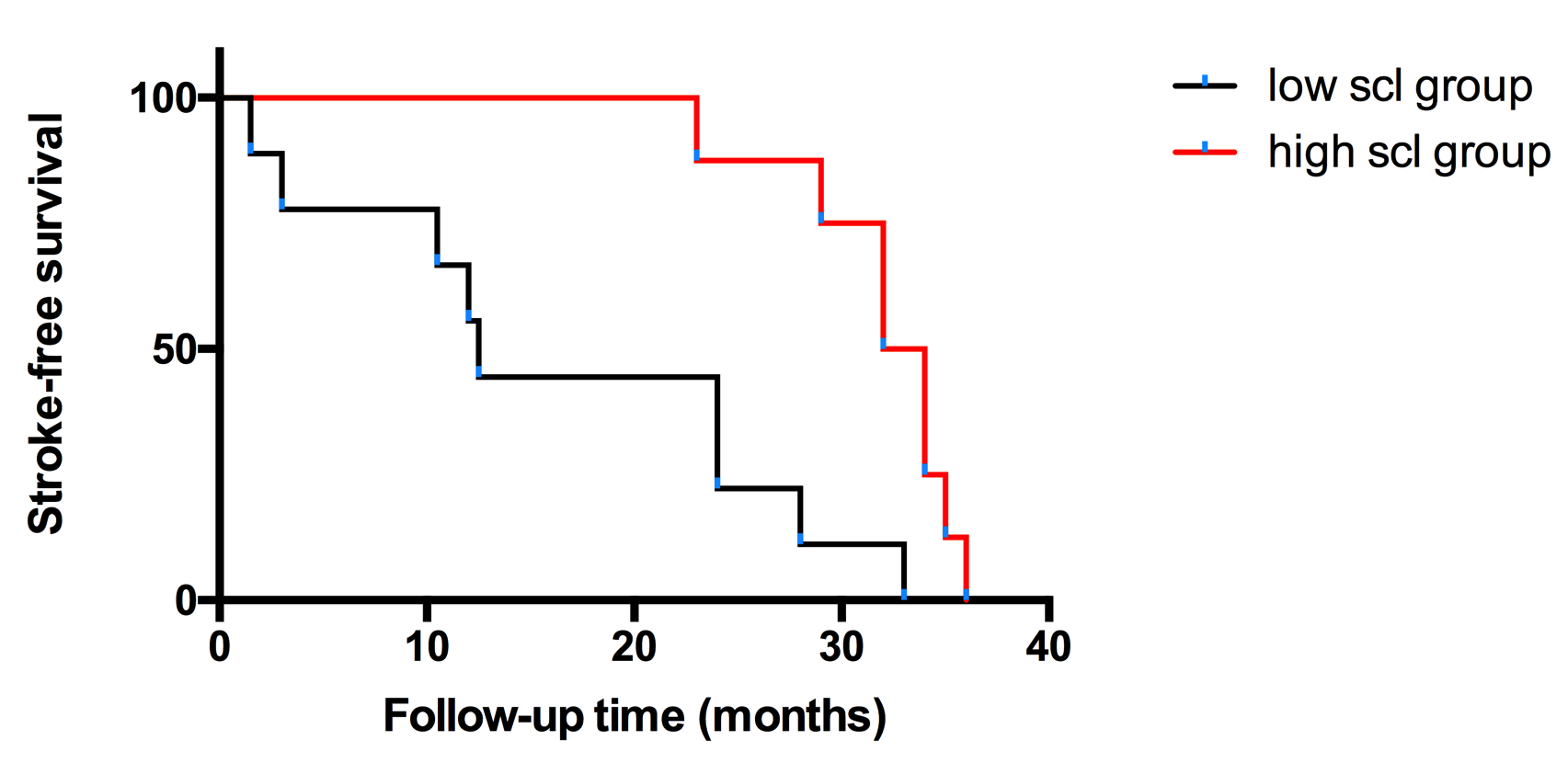


**Fig. 6** Kaplan-Meier survival curves of the Stroke-free rate for the low scl group and the high scl group(log-rank *p*=0.003).

**Table 4** The prognostic power of predictive models with or without sclerostin for adverse outcomes.

| Outcome | Predictive model with sclerostin | | Predictive model without sclerostin | |
| --- | --- | --- | --- | --- |
|  | C-index | AUC | C-index | AUC |
| MACCEs | 0.867(0.830-0.904) | 0.895 (0.854-0.936) | 0.857(0.818-0.896) | 0.888 (0.845-0.931) |
| All-cause mortality | 0.773(0.704-0.842) | 0.778 (0.704-0.852) | 0.713(0.642-0.784) | 0.723 (0.638-0.808) |
| MI | 0.856(0.793-0.919) | 0.870 (0.801-0.939) | 0.824(0.757-0.891) | 0.839 (0.762-0.915) |

The independent factors in the multivariate Cox model were further adopted into the predictive model for adverse outcomes. The predictive model for MACCEs includes sclerostin, the prevalence of osteoporosis, CCS class, frailty status, and the usage of statins; the predictive model for all-cause mortality includes sclerostin, the usage of beta blockers, and frailty status; the predictive model for MI includes sclerostin, PCI failure, frailty status, and the presence of multivessel disease.

**Abbreviation:** MACCEs, main adverse cardiac and cerebral events; MI, myocardial infarction; C-index, concordance index; AUC, area under the curve.
